# Electrospun Materials Based on Cellulose Acetate Loaded with Rosmarinic Acid with Antioxidant and Antifungal Properties

**DOI:** 10.3390/biomimetics9030152

**Published:** 2024-03-01

**Authors:** Mariya Spasova, Nikoleta Stoyanova, Olya Stoilova

**Affiliations:** Laboratory of Bioactive Polymers, Institute of Polymers, Bulgarian Academy of Sciences, Akad. G. Bonchev St., bl. 103A, BG-1113 Sofia, Bulgaria; nstoyanova@polymer.bas.bg

**Keywords:** electrospinning, cellulose acetate, rosmarinic acid, polyethylene glycol, antioxidant activity, *C. albicans*

## Abstract

Fibrous cellulose acetate (CA) materials loaded with rosmarinic acid (RA) were successfully created by one-pot electrospinning. In order to improve the water solubility of the polyphenolic acid and to facilitate its release from the fibrous materials, the non-ionic water-soluble polyethylene glycol (PEG) was added. Detailed characterization of the fabricated fibrous CA/RA and CA/PEG/RA materials was performed using scanning electron microscopy (SEM), X-ray diffraction analysis (XRD), UV-Vis spectroscopy and water contact angle analysis. The optimal ratio between CA, RA and PEG for preparation of defect-free and uniform fibers was accomplished by varying their concentrations. Furthermore, the incorporation of the PEG improved the hydrophilicity and wettability of the fibrous CA materials. Moreover, PEG facilitated the RA release and over 360 min, the amount released from fibrous CA/PEG/RA fibers was 91%, while that released from CA/RA materials was 53%. Both of the RA-containing fibrous materials, with and without PEG, manifested high antioxidant activity as determined by the DPPH free radical-scavenging method. In addition, the electrospun CA/PEG/RA materials displayed good antifungal activity against *C. albicans*. These features make the fibrous CA/PEG/RA materials promising candidates for treatment of wound infections.

## 1. Introduction

Since ancient times, nature has been a valuable source of plants for medicinal purposes [1]. Recently, interest in medicinal plants or their extracts has grown significantly, as they are more tolerable and less hazardous to the human body than synthetic ones and contain a set of biologically active compounds that resembles combined therapy with several synthetic components. This approach is one of the main approaches to overcoming multidrug resistance, the result of overuse of antibiotics and other drugs [2].

In recent years, interest in natural polyphenol compounds, which are often used in pharmacy and medicine for the prevention, prophylaxis and therapy of difficult-to-treat and socially significant diseases, has grown significantly [3]. This increasing interest in plant polyphenols is due to their abundance in foods, fruits, seeds, beverages and vegetables, the regular consumption of which is proved to be beneficial to human health. The general metabolism of polyphenols in plants results in the production of many phenolic acids, including gallic, ferulic, caffeic and rosmarinic acids [4]. It is known that they possess a number of valuable biological properties: antioxidant (eliminate free radicals to decrease inflammatory reactions and the aging impacts on cells), antibacterial, antitumor, anti-inflammatory, antiviral, etc. [5,6,7,8]. The effectiveness of these natural compounds depends on their water solubility and bioavailability. A disadvantage, however, is their instability during processing and storage, which limits their biological activity and potential health benefits. Their increased sensitivity to environmental factors, as well as their rapid oxidation, also significantly limit the possibilities for their local application.

Rosmarinic acid is a natural non-steroidal polyphenol antioxidant with significant biological properties such as antimicrobial, anti-inflammatory and anticancer properties [9,10]. A number of plants from the *Lamiaceae* family such as rosemary (*Rosmarinus officinalis*), Spanish sage tea (*Salvia lavandulifolia*), sage tea (*Salvia officinalis*), basil (*Ocimum tenuiflorum*), oregano (*Origanum vulgare*), marjoram (*Origanum majoroana*) and lemon balm (*Melissa officinalis*) are rich in rosmarinic acid [11,12]. The literature contains no proof that using rosmarinic acid is harmful. Its low bioavailability, which severely restricts its use as a therapeutic agent in clinical practice, is due to its limited solubility in water and bodily fluids, chemical instability, poor absorption, fast metabolism, and rapid elimination from the human body, despite its high therapeutic potential [13]. This necessitates the development of innovative biomaterials that can effectively and suitably deliver rosmarinic acid, hence increasing its bioavailability, or demands for the discovery of new, rational methods to enhance preexisting ones.

It is well known that delivery systems for biologically active molecules obtained by encapsulation technology are widely used to increase these compounds’ solubility, stability over time, and sustained release. One of the most studied systems and carriers of rosmarinic acid are polymer nanoparticles, due to the fact that they successfully preserve its biological properties [14,15,16]. There is a study in the literature revealing the fabrication of an edible film from a conjugate of rosmarinic acid with gelatin, which shows good physico-mechanical properties and a remarkable capacity to block UV light [17]. Complexation of rosmarinic acid with phospholipids to improve its oral bioavailability has also been reported [18]. There is also a report showing the incorporation of rosmarinic acid into cyclodextrin, which creates a complex that is soluble in water and has enhanced stability, solubility and bioavailability [19].

Recently, in addition to polymer nanoparticles, fibrous polymer materials (so-called non-woven materials) produced by electrospinning have attracted a lot of attention as rosmarinic acid carriers [20]. The electrospinning method allows relatively easy fabrication of fibrous materials made of continuous polymer micro- and nanofibers with a controlled diameter. The resulting polymer materials have unique characteristics, including a high specific surface area, porosity, low weight and the potential for simple functionalization. A number of articles show that this method allows the effective loading of various biologically active substances with low molecular weight, as well as the control of their release profile [21,22,23]. For this reason, encapsulating polyphenol compounds in nanofibers by electrospinning or nanoparticles by electrospraying has recently been the focus of several investigations [24].

Up to now, there have been reports of fibrous materials produced by electrospinning from poly(ε-caprolactone) solutions containing magnetite and rosmarinic acid that may be used as drug delivery systems [25]. Additionally, a study in the literature demonstrated that loading of rosemary extract into polyvinyl alcohol fibers by electrospinning resulted in antioxidant activity [26]. Furthermore, electrospinning of cellulose acetate containing rosmarinic acid in concentrations of 5 and 10% has been reported as well [27]. Unfortunately, in this work, a major downside is the usage of a very low solution feed rate, which results in a much longer time taken to create the non-woven materials.

As far as we are aware, the fabrication of fibrous mats by one-pot electrospinning of a solution containing cellulose acetate (CA), rosmarinic acid (RA) and polyethylene glycol (PEG) has not been reported yet. Herein, the non-ionic water-soluble PEG was added to improve the solubility in water of the RA and to facilitate RA release from the fibrous CA materials. By varying the concentrations of spinning solution, the ratio between CA, RA and PEG, as well the process conditions (applied voltage, needle tip-to-collector distance and flow rates), uniform and defect-free CA/RA and CA/PEG/RA fibrous materials were fabricated. Finally, the antioxidant and antifungal activities against *C. albicans* were evaluated to show the potential of the obtained fibrous materials loaded with RA as effective novel wound-healing dressings.

## 2. Materials and Methods

### 2.1. Materials

Cellulose acetate (CA, 30,000 g/mol and acetyl content 39.8%), rosmarinic acid (RA, 96%), ethanol (≥99.8% GS), acetone and 2,2-diphenyl-1-picrylhydrazyl (DPPH) were purchased from Sigma-Aldrich (Darmstadt, Germany). Polyethylene glycol (PEG, 1900–2200 g/mol) was supplied by Fluka (Buchs, Switzerland), while Tween 80—by Acros Organics (Geel, Belgium). All reagents were of analytical-grade purity and were used as received.

### 2.2. Fabrication of the Electrospun Fibrous Materials

Fibrous CA, CA/PEG, CA/RA and CA/PEG/RA fibrous mats were prepared by an electrospinning process. By varying the concentration of RA in spinning solutions, it was found that the maximum amount that can be incorporated into fibrous CA materials without hindering the electrospinning process is 20 wt% (in respect to total polymer weight). The CA spinning solution was prepared as previously described in [7], namely by dissolving the CA in a mixed acetone/water solvent at 80/20 *v/v* and concentration of 10 wt%. In addition, the following spinning solutions were prepared in acetone/water 80/20 *v*/*v*: CA/PEG (80/20 *w*/*w*); CA with RA (20 wt% in respect to the CA weight); and CA/PEG (80/20 *w*/*w*) with RA (20 wt% in respect to the total polymer weight). In all mixed spinning solutions, the total polymer concentration was kept at 10 wt%. Then, spinning solutions were loaded into a 5 mL plastic syringes capped with a 20-gauge needle, connected to the positively charged electrode. The electrode was connected to a specially designed high-voltage power source that could provide positive DC voltage in the 10–30 kV range. The 45 mm diameter grounded rotating collector was positioned 15 cm from the needle tip, and its rotational speed was maintained at 1000 rpm. The spinning solution was fed at a constant rate using a horizontally positioned infusion pump (NE-300, New Era Pump Systems Inc., Farmingdale, NY, USA). Electrospinning was performed at a constant applied voltage of 25 kV, at a constant feed rate of 3 mL/h, at an ambient temperature of 21 °C and at relative humidity of 50%. In order to eliminate any solvent residues, all electrospun materials were dried under a vacuum (25 °C).

### 2.3. Complex Characterization of the Fabricated Electrospun Materials

The fabricated CA-based fibrous materials were thoroughly studied morphologically using scanning electron microscopy (SEM). All of the mats were vacuum-coated with gold for 40 s using a Jeol JFC-1200 fine coater before being observed on a Jeol JSM-5510 (JEOL Co., Ltd., Tokyo, Japan). Using Image J 1.54g software, at least 30 fibers from SEM images were measured to determine the mean fiber diameter and the standard deviation [28]. A Brookfield DV-II+ Pro programmed viscometer (Brookfield Engineering Laboratories, Middleboro, MA, USA) fitted with a sample thermostatic cup and a cone spindle running at 25 °C was used to measure the dynamic viscosities of the spinning solutions.

In order to evaluate the crystalline structure of the prepared CA-based fibrous materials, X-ray diffraction analysis (XRD) was applied. With a filtered CuKα radiation source and a bright detector, a D8 Bruker Advance powder diffractometer (Bruker, Billerica, MA, USA) was used to record the XRD patterns in the 2θ range of 10° to 60° (step of 0.02° and counting time of 1 s/step).

Based on the surface wettability properties, the hydrophobic–hydrophilic properties of the CA-based electrospun mats were measured using an Easy Drop DSA20E Krűss GmbH apparatus (Hamburg, Germany). Computer analysis was used to calculate the average contact angle value after a sessile drop of deionized water (10 μL) was dropped onto the surface of fibrous samples (2 cm × 7 cm cut in the direction of the collector rotation). Every sample was subjected to 10 measurements.

The amount of RA loaded in CA fibrous materials was determined by dissolving the fibrous CA samples (1 cm^2^) in acetone_80_/water_20_ (10 mL) followed by measuring the absorbance at 321 nm using a UV-Vis spectrophotometer (DU 800, Beckman Coulter, Brea, CA, USA). The release profile of RA from fibrous CA materials was examined in vitro at 37 °C in a phosphate-buffered solution (pH 7.4, ionic strength 0.1) in the presence of Tween 80 (buffer/Tween 80 = 99.2/0.8 *v*/*v*). The samples (15 mg) were immersed in 100 mL of the buffer solution, placed in a shaking thermostatic water bath (Julabo SW23, Allentown, PA, USA) and stirred at 150 rpm. At certain intervals, aliquots were taken and the absorbance at 321 nm was measured. The same amount of fresh buffer solution was added back. Using calibration curves in phosphate buffer/Tween 80 (99.2/0.8 *v*/*v*) with a correlation coefficient of R = 0.999, the quantity of RA released over time was determined.

### 2.4. Antioxidant Activity Determination

The 2,2-diphenyl-1-picrylhydrazyl (DPPH) free radical-scavenging method was used to assess the antioxidant activity of the fibrous CA materials. Briefly, 0.5 mL of RA solution in ethanol (5 × 10^−3^ M) or fibrous CA/PEG, CA/RA and CA/PEG/RA samples (0.5 mg) were added to a 3 mL of DPPH solution (1 × 10^−4^ M) in ethanol. Then, as-prepared samples were stored in the dark at 20 °C for 30 min. In order to determine the amount of DPPH radicals remaining in the solution, the absorbance at 517 nm was measured using a UV-Vis spectrophotometer (DU 800, Beckman Coulter, CA, USA). The antioxidant activity (AA%) was calculated by the following equation:

$$\mathrm{AA}\%=\left[\frac{\left(A_{DPPH}-A_{sample}\right)}{A_{DPPH}}\right]\;\times \;100\;,$$

where A_DPPH_ is the absorbance of DPPH• solution at 517 nm and A_sample_ is the absorbance of DPPH• solution at 517 nm in the presence of fibrous CA materials. The experiments were repeated three times.

The 2,2′-azino-bis(3-ethylbenzothiazoline-6-sulfonic acid) (ABTS) radical cation scavenging method was also used to assess the total antioxidant activity of the fibrous CA materials. In particular, an aqueous stock solution (7 mM) of ABTS was prepared. The ABTS radical cation was produced by reacting this stock solution with 2.45 mM aqueous solution of K_2_S_2_O_8_. Then, the mixed solution was kept in the dark for 16 h at room temperature in order to react the components. Prior to use, the blue-green solution was diluted with ethanol to give an absorbance of a minimum of 0.700 at 734 nm in a 1 cm cuvette. Samples (0.5 mg) of fibrous CA, CA/PEG, CA/RA and CA/PEG/RA materials were immersed in 3 mL of blue-green ABTS solution in ethanol. Then, as-prepared samples were stored in the dark at room temperature for 5 min. The absorbance decrease was monitored at 734 nm by a UV-Vis spectrophotometer (DU 800, Beckman Coulter, CA, USA). The antioxidant activity (AA%) was calculated by the following equation:

$$\mathrm{AA}\%=\left[\frac{\left(A_{ABTS}-A_{sample}\right)}{A_{ABTS}}\right]\;\times \;100\;$$

where A_ABTS_ is the absorbance of ABTS•+ solution at 734 nm and A_sample_ is the absorbance of ABTS•+ solution at 734 nm in the presence of fibrous CA materials. The radical stock solution was freshly prepared, and all experiments were performed in triplicate.

### 2.5. Antifungal Properties of the Fabricated Electrospun Materials

The antifungal activity of the prepared electrospun mats was tested against *C. albicans* 74 that were purchased from the National Bank for Industrial Microorganisms and Cell Cultures (NBIMCC, Sofia, Bulgaria). The antifungal activity of the fibrous CA samples against the used fungi was evaluated by a viable cell-counting method. Following a 30 min UV sterilization, the samples (~5 mg) were in contact with a 3 mL fungal suspension (106 cells/mL) that had been made from nutritional broth (Sigma-Aldrich, Darmstadt, Germany) at 37 °C. Aliquots (50 mL) were taken at 4 and 24 h, and they were diluted ten times with sterile phosphate-buffered saline (PBS). After that, they were placed on Petri dishes with nutrient agar (Sigma-Aldrich, Darmstadt, Germany) and then the plates were incubated at 37 °C for 24 h. The number of surviving fungi was evaluated by counting the colony-forming units (CFU) in triplicate for each experiment. A study of the fungal adhesion to the surface of the fibrous samples was performed by direct SEM observation with Jeol JSM-5510 (Jeol Ltd., Tokio, Japan). In brief, the fibrous samples were incubated in 2 mL of broth culture of *C. albicans* 74 with a concentration of 10^6^ cell/mL for a time period of 72 h. After that, the samples were washed twice with PBS (pH 7.4) in order to remove the non-adhered cells. After carefully washing the mats in PBS for five hours at 4 °C with a 2.5 wt% glutaraldehyde solution, the adhering fungi were fixed. This was followed by freeze-drying, gold coating, and SEM evaluation.

### 2.6. Statistical Data Analysis

The current investigation’s findings were presented as means ± standard deviation (SD). One-way analysis of variance (ANOVA) and the post hoc comparison test (Bonferroni) were used to determine the statistical significance of the data. GraphPAD Prism software, version 5 (GraphPad Software Inc., San Diego, CA, USA), was used for this study. The following results were deemed statistically significant: * *p* < 0.05, ** *p* < 0.01, and *** *p* < 0.001.

## 3. Results and Discussion

The proposed approach aims to combine the biocompatible CA and water-soluble PEG with low-toxicity as suitable carriers of a natural polyphenol compound RA with antioxidant, anticancer and antimicrobial activities with potential biomedical application. In this way, the appropriate encapsulation of the bioactive substance in these selected polymers will enhance its water solubility and bioavailability and will preserve its activity via storage.

**Figure 1:**
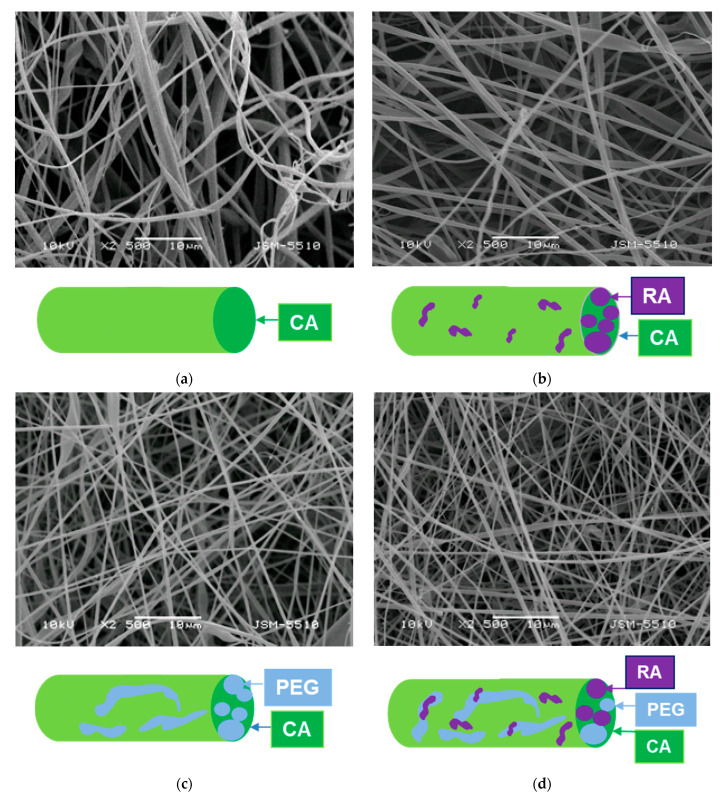
SEM micrographs of the non-woven mats and schematic illustration of the cross-section of fibers based on: CA (**a**), CA/RA (**b**), CA/PEG (**c**) and CA/PEG/RA (**d**).

Diverse types of non-woven mats from nontoxic, nonirritant, and biodegradable CA, water-soluble PEG and a polyphenol compound RA were fabricated by one-pot electrospinning. SEM micrographs and schematic presentation of the fabricated fibrous CA, CA/PEG, CA/RA and CA/PEG/RA electrospun mats were presented in Figure 1. It is evident that the electrospinning parameters used led to the production of homogeneous CA fibers. It is interesting to observe that the mean fiber diameter varied according to the spinning solutions’ composition. Continuous fibers with a mean fiber diameter of 780 ± 100 nm were produced by electrospinning the CA solution (Figure 1a). Adding RA to the CA solution led to a slight decrease in the mean fiber diameter to 670 ± 70 nm. Moreover, the average diameters of CA/PEG and CA/PEG/RA fibers (Figure 1c,d) were 370 ± 40 nm and 360 ± 35 nm, respectively. Apparently, this decrease in the mean diameters was probably due to the adding of the water-soluble PEG, which possesses a low molecular weight. Thus, the addition of PEG into the spinning solution led to a decrease in its viscosity. To prove this, the dynamic viscosities of CA, CA/RA, CA/PEG and CA/PEG/RA solutions were measured, and their values were 185, 192, 90 and 94 cP, respectively. Therefore, the addition of PEG resulted in a more significant decrease in the dynamic viscosities of the spinning solutions and resultant mean fibers diameter than those of RA, which is in accordance with the literature [29].

**Figure 2:**
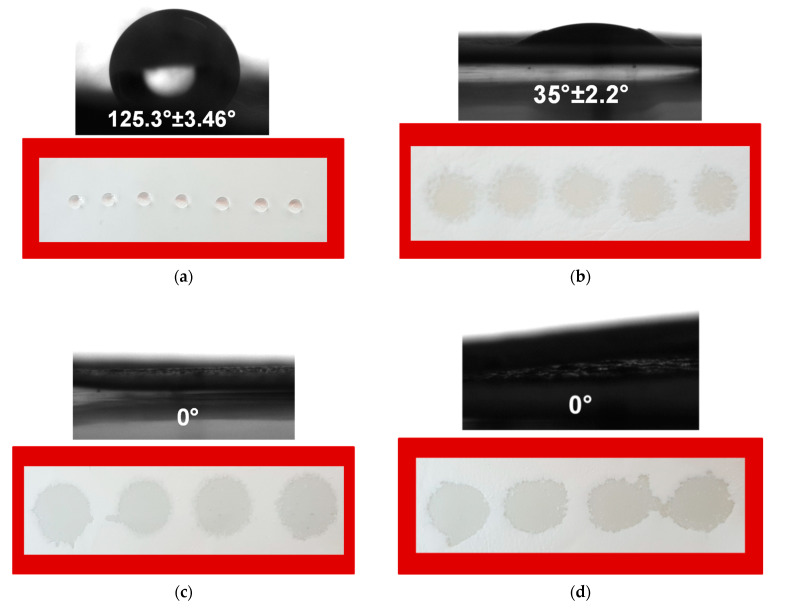
The contact angle values and digital photographs of water droplets poured onto non-woven materials of CA (**a**), CA/RA (**b**), CA/PEG (**c**) and CA/PEG/RA (**d**). The volume of the water droplet was 10 μL.

The ability to develop, as well to control, the surface and volume behavior of the used materials is crucial for pharmaceutical, medical and cosmetic applications. In this regard, a material surface that absorbs fluids rapidly can also release integrated bioactive substances more rapidly. In the initial minutes or hours, this will cause a burst release. Conversely, surfaces made of more hydrophobic materials will result in an initial delay in the medication’s release and diffusion of the liquid media. To regulate the release behavior, it is crucial to measure the contact angle of the resulting electrospun mats and to discover the surface’s hydrophilic–hydrophobic equilibrium. As a result, the resulting non-woven materials’ water contact angle values were determined, and the shape of the water droplet that was deposited on their surfaces was recorded. Figure 2 displays the observed values of the water contact angle along with digital photographs of water droplets deposited onto the surface of CA, CA/RA, CA/PEG and CA/PEG/RA fibrous materials. Obviously, the surface of the CA fibrous mat was hydrophobic with a water contact angle value of around 125°. The shape of the deposited droplets on the CA surface was round and did not spread (Figure 2a). The fibrous CA/RA materials were hydrophilic with a value of the water contact angle of 35° (Figure 2b). This decrease in the contact angle was due to the addition of RA, which possesses some slight solubility in water (18 mg/mL). A significant decrease in the water contact angle value was detected by adding the water-soluble PEG to the spinning solution and, respectively, to the composition of the non-woven materials. This increase in wettability is well described in the literature [30]. Both electrospun CA/PEG and CA/PEG/RA non-woven materials were superhydrophilic, possessing water contact angle values of 0°. When it came into contact with the surface of the mat, the water droplet spread immediately into the materials’ volume and surface. As can be clearly seen, the droplets tended to coalesce.

**Figure 3:**
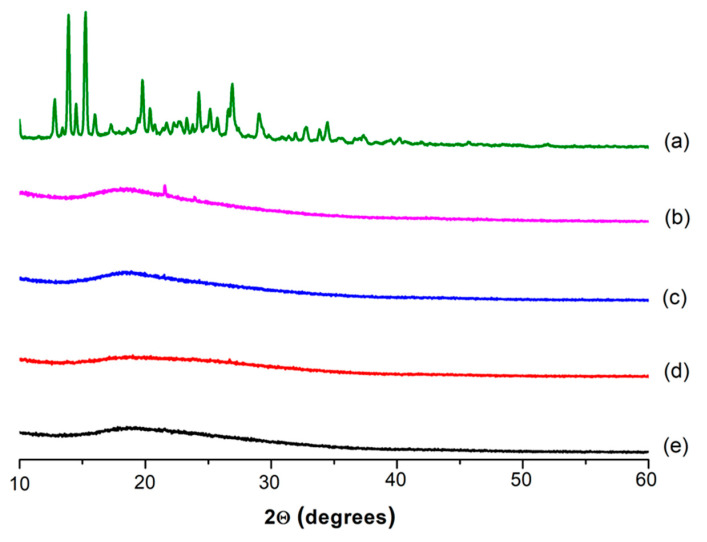
XRD patterns of (**a**) RA (powder) and fibrous (**b**) CA; (**c**) CA/PEG; (**d**) CA/RA and (**e**) CA/PEG/RA materials.

Figure 3 shows the XRD patterns of the RA and fibrous CA, CA/PEG, CA/RA and CA/PEG/RA materials. As described in the literature, the RA (powder) revealed a significant degree of crystallinity across the experimental 2θ range, with prominent peaks [31,32]. As shown, RA showed diffraction peaks at 13.7, 15.2, 19.8 and 27.0, which confirms its crystallinity. In contrast, the subjection of CA and CA/PEG solutions to electrospinning resulted in preparation of non-woven materials with amorphous structures. This finding is consistent with related results in the literature [33]. Interestingly, the incorporation of the polyphenol compound in the fibrous materials obtained by electrospinning resulted in the preparation of novel materials with amorphous structures as well. This is most likely due to the specificity of the electrohydrodynamic electrospinning process, where the rapid solvent evaporation during the process does not provide enough time for the bioactive agent to form a crystal lattice. In this way, the crystalline RA loaded into the spinning solution remains in its amorphous form during the electrospinning, which is highly desirable from the biomedical application point of view.

**Figure 4:**
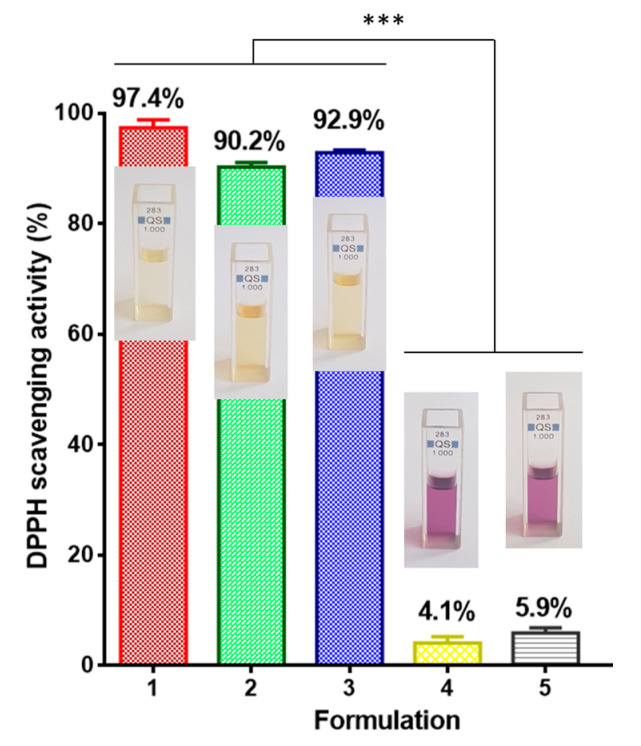
Antioxidant activity of: RA ethanol solution (1); and fibrous CA/RA (2); CA/PEG/RA (3); CA (4); CA/PEG (5) materials. *** *p* < 0.001. Digital photographs of the solutions are displayed as well.

Reactive oxygen species accumulation over time results in oxidative stress in cells, which is a major factor in the induction and progression of diverse illnesses [34]. Therefore, in recent years, the therapeutic approach has mainly focused on the use of antioxidants of plant origin. It is known that RA exerts a powerful antioxidant effect [35]. Therefore, it was of interest to study the antioxidant properties of the prepared novel fibrous materials loaded with RA. The DPPH● scavenging activity of CA and CA/PEG materials containing RA was monitored spectrophotometrically by recording the absorbance of DPPH● at 517 nm for 30 min after contact with the fibrous samples. In addition, the DPPH scavenging ability of CA and CA/PEG fibrous materials was established for comparison. The results obtained from the antioxidant activity were presented in Figure 4. As shown, the neat polymer fibrous materials had an insignificant effect on the DPPH solutions. After contact with the CA and CA/PEG materials, the absorbance of DPPH● was reduced by ~4% and 5.9%, respectively. In addition, the DPPH solution color was not visibly altered and remained purple. In contrast, after contact with the non-woven materials loaded with RA the DPPH solution changed its color to pale yellow. Moreover, the measured absorbance of DPPH● decreased by 90.2% and 92.9% after 30 min contact with electrospun CA/RA and CA/PEG/RA materials. Undoubtedly, the novel materials exhibited excellent antioxidant activity. Furthermore, the change in DPPH solution absorption following contact with RA ethanol solution is comparable to that observed upon contact with RA-containing fibrous materials at the same bioactive component concentration. It is observable that the fibrous materials loaded with RA and PEG demonstrated slightly higher antioxidant activity than mats of CA/RA that could be attributed to the more burst release polyphenol compound from the CA/PEG/RA mats. The obtained results indicated that RA loaded in the CA and CA/PEG non-woven materials had preserved its high antioxidant activity.

In fact, ABTS and DPPH tests are known as mixed tests, including the transfer of both a hydrogen atom and an electron. For that reason and in order to confirm the results of DPPH assay, an additional ABTS assay was performed. According to Figure S1, fibrous CA/RA and CA/PEG/RA materials displayed high antioxidant activity of 86.1% and 93.3%. Moreover, a change of color from blue-green to pale yellow was also observed. In contrast, the scavenging capacity of fibrous CA and CA/PEG materials was very low—around 2.4% and 3.2%, respectively. The solution color was not visibly altered and remained blue-green. Evidently, RA preserved its strong oxidizing activity after loading in fibrous CA and CA/PEG materials.

**Figure 5:**
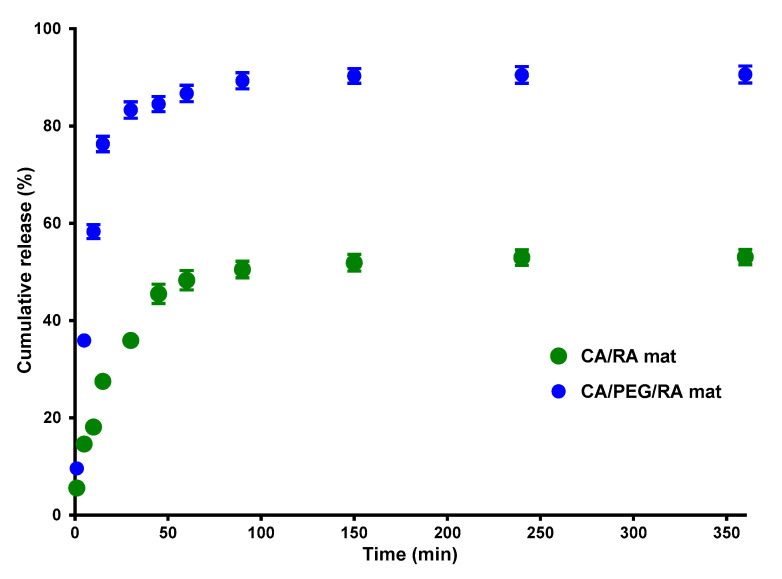
In vitro RA release profile from non-woven CA/RA and CA/PEG/RA materials in phosphate-buffered solution with pH 7.4. The average values from three different measurements are shown together with the corresponding standard deviation to represent the results.

The RA release from the non-woven CA/RA and CA/PEG/RA materials was measured spectrophotometrically, and the release profile is presented in Figure 5. Initially, up to 60 min, a burst release of the RA from the fibrous materials was observed. After that, within 360 min, a gradual mode of the RA release was detected, followed by reaching a plateau. As expected, the PEG addition facilitated the RA release and over 360 min, the amount released from the fibrous CA/PEG/RA mats was 91%, while that released from the CA/RA mats was 53%. This clearly shows that the amount and the rate of the released RA from fibrous CA materials depends on the presence of the water-soluble polymer PEG. This is consistent with other reports on the effect of the incorporation of water-soluble polymer in the fibers on the increased release rate of the drug [36].

**Figure 6:**
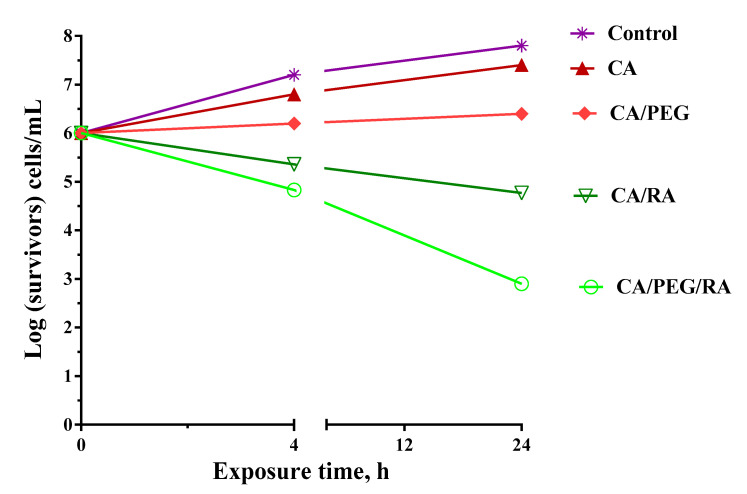
The number of viable *C. albicans* cells versus the exposure time shown logarithmically.

Highly porous polymer materials such as nano sponges [37,38] and nanofibers [39] have been proved as good delivery devices for antifungal drug delivery. In view of the potential biomedical application of the prepared materials for the treatment of fungal infections in burn wounds, the antifungal activity of the prepared fibrous materials against *C. albicans* was evaluated by direct counting of the viable fungi after incubation (4 h and 24 h) with non-woven materials. The amount of fungus that survived was then determined by plating and counting CFU in a solid medium. Figure 6 presents the log of the surviving fungi vs. the exposure time for the prepared materials. For sake of comparison, the growth of *C. albicans* control was determined as well. It was discovered that during the experiment, the fungal control grew normally. As seen (Figure 6), the fibrous CA and CA/PEG materials do not alter the fungal growth, and their number after 24 h is more than 6 log. However, a reduction in the number of viable pathogen cells that were in contact with RA-containing fibrous materials was observed even after 4 h. Moreover, a significant reduction in fungal number was detected at 24 h, especially for the non-woven CA/PEG/RA materials, and the *C. albicans* titer decreased by six and three orders of magnitude. This might be explained by the fibrous composition containing a water-soluble polymer that assists the release of the natural polyphenol and supports the manifestation of its antifungal action.

**Figure 7:**
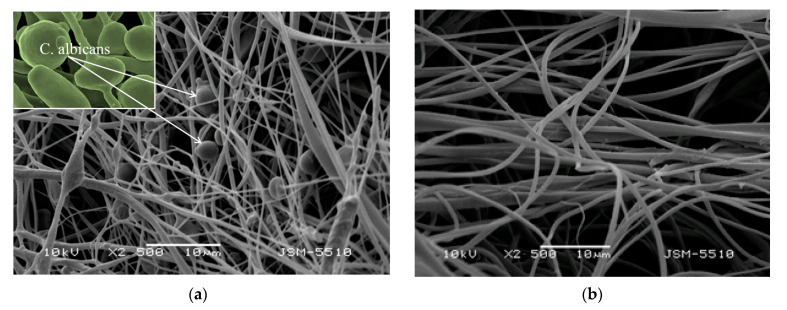
SEM micrographs of electrospun CA/PEG (**a**) and CA/PEG/RA (**b**) materials after 72 h incubation in *C. albicans* cell culture.

It is well known that *C. albicans* is able to form biofilms that can cause invasive systemic infections of tissues and organs. The first crucial step of biofilm formation begins with yeast cells adhering to a certain surface and then forming a distinct colony. For that reason, in the present study the adhesion of *C. albicans* cells onto the surface of the prepared non-woven materials was observed by SEM analysis. The samples were examined after incubation in a fungal suspension (10^6^ cells/mL) for 72 h at 37 °C. Figure 7 presented the SEM micrographs of CA/PEG and CA/PEG/RA fibrous materials after incubation in *C. albicans* cell culture. Obviously, *C. albicans* cells adhered to the surface of the fibrous CA/PEG materials (Figure 7a). Moreover, the pathogenic cells retained their characteristic morphology with an oval shape and a size of about 4 µm. In contrast, there was a tendency toward prevention of the fungal adhesion and growth on the RA-containing fibrous materials (Figure 7b) due to the antifungal activity of RA loaded into the fibers. This finding, along with the results from the number of viable pathogenic cells, revealed and proved that RA-containing materials exhibit antifungal activity.

## 4. Conclusions

In this study, the preparation of electrospun CA and CA/PEG electrospun mats incorporated with natural polyphenol compound—rosmarinic acid was reported for the first time. The performed XRD analysis revealed that the RA incorporated into the non-woven materials is in an amorphous state, which is more favorable for its utilization in dosage forms. The presence of water-soluble PEG favors wetting and RA release from the electrospun materials. The CA/RA and CA/PEG/RA materials were effective in inhibiting the growth of the fungi *C. albicans*. Furthermore, the fibrous materials containing RA exhibited the potential to inhibit pathogenic cell adhesion and biofilm development. In addition, the novel fibrous materials loaded with the natural polyphenol compound exhibited high antioxidant activity (almost 93%). Therefore, the fabricated fibrous materials containing the phenol compound RA with multiple bioactivities are promising candidates for use in wound healing.

## Data Availability

Data are contained within the article and Supplementary Materials.

## Supplementary Materials

The following supporting information can be downloaded at: https://www.mdpi.com/article/10.3390/biomimetics9030152/s1, Figure S1: Antioxidant activity determined by ABTS method of: RA ethanol solution (1); and fibrous CA/RA (2); CA/PEG/RA (3); CA (4); CA/PEG (5) materials. *** *p* < 0.001. Photos of the corresponding solutions are shown as digital images.
