# Supplementary material for: Electrospun Materials Based on Cellulose Acetate Loaded with Rosmarinic Acid with Antioxidant and Antifungal Properties
Source: Biomimetics (Basel). 2024 Mar 1;9(3):152. doi: 10.3390/biomimetics9030152 (PMC10968000; doi:10.3390/biomimetics9030152)
Supplement: Supplementary file 1 [file biomimetics-09-00152-s001.zip › biomimetics-2839150-supplementary.pdf]

Supplementary Material

Electrospun materials based on cellulose acetate loaded with rosmarinic acid with antioxidant and antifungal properties

Mariya Spasova*, Nikoleta Stoyanova and Olya Stoilova*

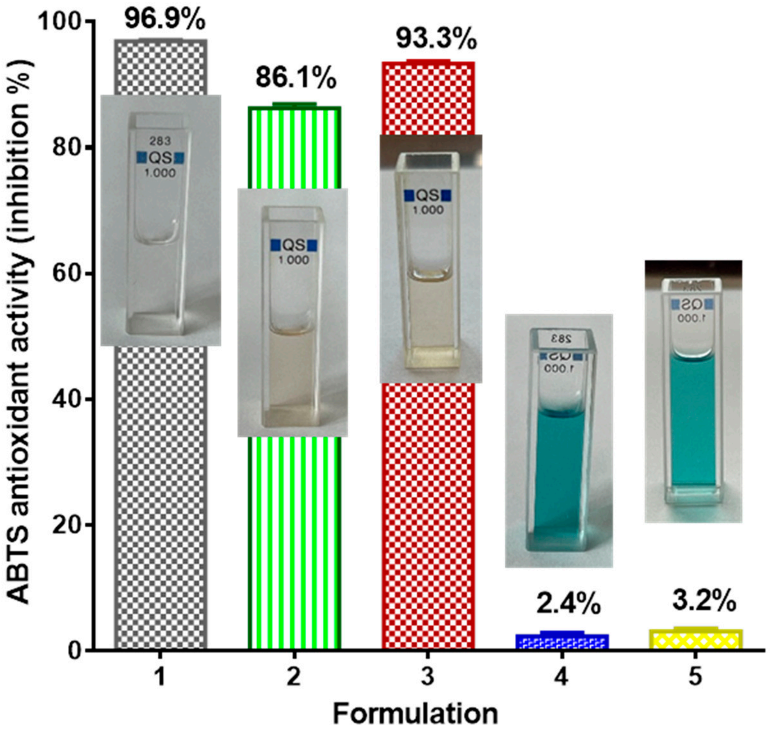

Figure S1. Antioxidant activity determined by ABTS method of: RA ethanol solution (1); and fibrous CA/RA (2); CA/PEG/RA (3); CA (4); CA/PEG (5) materials. ***p<0.001. Photos of the corresponding solutions are shown as digital images.
